# Ipilimumab and nivolumab for cancer treatment: a pharmacovigilance study based on the FDA adverse event reporting system database

**DOI:** 10.3389/fendo.2026.1786504

**Published:** 2026-06-18

**Authors:** Ruming Liu, Yaoyu Xiang, Xidan Hu, Min Zhang, Lujie Wang, Tao Liu, Xi Wang, Bin Qian

**Affiliations:** 1 Department of Clinical Pharmacy, The First Affiliated Hospital of Kunming Medical University, Kunming, Yunnan, China; 2 Department of Sports Medicine, The First Affiliated Hospital of Kunming Medical University, Kunming, Yunnan, China; 3 Organ Transplantation Center, The First Affiliated Hospital of Kunming Medical University, Kunming, Yunnan, China

**Keywords:** cancer, disproportionality analysis, FDA adverse event reporting system, ipilimumab, nivolumab

## Abstract

**Background:**

Cancer remains one of the leading is associated with of death globally. This study analyzed adverse drug event (ADE) signals and potential factors associated with reporting in ipilimumab and nivolumab-treated cancer data from the Food and Drug Administration’s (FDA) Adverse Event Reporting System (FAERS) database.

**Methods:**

This study collected ADEs associated with ipilimumab, nivolumab, and their combination therapy for all cancers from the first quarter of 2004 to the fourth quarter of 2024 through the FAERS database. It investigated the basic information of all adverse reaction reports, analyzed the cumulative trends of ADE onset time across different age groups, and mined safety signals significantly associated with the three drug groups via disproportionality analysis. Additionally, the study explored potential factors associated with reporting of these adverse reactions.

**Results:**

A total of 21,712,563 reports were included in this study as research subjects, among which there were 4,600, 36,556, and 10,862 adverse reaction reports for ipilimumab, nivolumab, and ipilimumab/nivolumab, respectively. Separate analysis of these three groups of drugs showed that the onset time of adverse reactions mostly exhibited a bimodal pattern characterized by “early concentrated outbreak + long-term persistent risk”, which was related to age. These three groups of drugs showed a stronger associated with colitis, adrenal insufficiency, malignant neoplasm progression, death, and intentional product use issues. Age and weight were identified as potential factors associated with reporting of adverse reactions related to these three groups of drugs.

**Conclusion:**

Based on the FAERS database, this study identifies significant pharmacovigilance signals and potential reporting-associated factors for ipilimumab, nivolumab, and their combination. Importantly, these findings represent hypothesis-generating signal detections rather than definitive causal risk estimations, underscoring the absolute necessity for heightened clinical vigilance and routine endocrinological monitoring. These findings inform clinical monitoring strategies and provide a foundation for future research into the molecular potential mechanisms underlying irAEs.

## 1 Introduction

Cancer is defined as a pathological condition manifested through the deregulated expansion of malignant cells, which undergo evolutionary processes governed by natural selection (1). This disease state typically involves aberrant cellular multiplication and impairment of replicative senescence mechanisms (2). Globally, the year 2022 witnessed nearly 20 million newly diagnosed cancer cases (incorporating nonmelanoma skin cancers) concurrent with 9.7 million cancer-associated fatalities (also inclusive of NMSC). Epidemiological projections indicate that roughly one in five individuals across all genders may receive a cancer diagnosis during their lifetime, while mortality statistics approximate one in nine males and one in twelve females succumb to the disease (3). Conventional oncological interventions—comprising surgical resection, chemotherapeutic regimens, and radiotherapy—continue to dominate clinical practice, notwithstanding recent extensive investigation into advanced modalities such as genetic and immunotherapeutic approaches (4). These treatment strategies, however, are frequently compromised by limitations including pharmacological resistance, significant adverse reactions, constrained applicability, and potential mutagenic consequences (4).Immunotherapy has established itself as a fundamental pillar in oncology following the 2011 regulatory approval of ipilimumab, the inaugural immune checkpoint blockade agent (5). Nivolumab and ipilimumab exhibit mechanistically distinct yet synergistic profiles: nivolumab facilitates the functional restoration of anti-neoplastic T-cells, whereas ipilimumab promotes *de novo* anti-tumor T-cell responses (6). Clinical investigations in advanced melanoma demonstrate that both nivolumab-ipilimumab combination therapy and nivolumab monotherapy produce sustained and enhanced therapeutic efficacy compared to ipilimumab alone; comparative analyses further establish the superior clinical outcomes associated with combination therapy (7).

Long-term follow-up data reveal that among patients discontinuing immunotherapy for ≥3 years, nivolumab-ipilimumab combination significantly improved 5-year survival rates versus chemotherapy, demonstrating durable clinical efficacy independent of tumor PD-L1 status. These findings substantiate the combination regimen as a primary therapeutic option for metastatic non-small-cell lung carcinoma (8).

Notably, combination therapy with ipilimumab and nivolumab is associated with significant immune-related toxicities, including thyrotoxic crisis, diabetic manifestations, immune-mediated optic neuritis, cystitis, eyelid ptosis, and complete atrioventricular block (9); in advanced melanoma management, this therapeutic combination correlates with an expanded spectrum of immune-mediated adverse events (10). Given the escalating global cancer incidence and evolving risk factors, combinatorial pharmacotherapy has emerged as a pivotal treatment paradigm (11). Systematic investigation of treatment-emergent adverse events across three therapeutic categories provides critical foundation for clinical surveillance and molecular mechanistic research in oncology.

The FDA Adverse Event Reporting System (FAERS) constitutes a comprehensive, publicly accessible pharmacovigilance database that aggregates spontaneous post-marketing reports of adverse events, product quality concerns, and medication errors for FDA-approved therapeutics (12, 13). While pharmaceutical manufacturers maintain mandatory reporting obligations, healthcare professionals and consumers worldwide contribute voluntarily. This spontaneous surveillance mechanism plays an indispensable role in pharmacovigilance, enabling safety signal detection and addressing inherent clinical trial limitations including restrictive designs, selective enrollment, limited cohort sizes, and abbreviated follow-up durations (14).As one of the most extensive global pharmacovigilance repositories (15), FAERS encompasses longitudinal data from 2004 to present, exceeding 20 million case entries. The database undergoes quarterly real-time updates, ensuring exceptional data transparency and accessibility (16). Although prior FAERS-based analyses have characterized adverse event profiles for ipilimumab-nivolumab combination therapy (9, 17), a comparative safety assessment of monotherapy versus combination regimens remains uninvestigated. The present study delivers a comprehensive safety evaluation of cancer immunotherapeutic agents, facilitating treatment optimization and providing substantive insights for future oncological drug safety monitoring and investigation.

This research undertook a systematic analysis of adverse event reports for three therapeutic categories (ipilimumab monotherapy, nivolumab monotherapy, and combination regimen) utilizing FAERS data spanning Q1–2004 through Q4 2024. Methodologically, we initially characterized baseline demographic and clinical profiles for each cohort, subsequently examining age-stratified cumulative incidence trends of adverse reactions. Four distinct algorithmic approaches were employed at the Preferred Term level to detect safety signals across the three therapeutic groups. Conclusively, we identified risk determinants for treatment-emergent adverse events, thereby establishing a foundation for enhanced clinical monitoring and elucidating molecular potential mechanisms underlying these oncological interventions.

## 2 Materials and methods

### 2.1 Data origin and study design

The original data of this study were sourced from the United States FDA FAERS (https://fis.fda.gov/extensions/FPD-QDE-FAERS/FPD-QDE-FAERS.html). This database is used to collect reports of ADEs spontaneously submitted by medical staff, patients, etc. It is updated quarterly, publicly available for free, and stored in the form of ASCII or XML. It is often used for the mining of ADE signals of drugs that have been launched on the market. The FAERS data files are composed of seven types of datasets: patient demographics and management information (DEMO), drug/biologic information (DRUG), adverse events (REAC), patient outcomes (OUTC), reporting source (RPSR), start and end dates of drug therapy (THER), and indications for use/diagnosis (INDI), as well as deleted cases. FAERS is a publicly accessible and anonymized database, for which the requirements of Institutional Review Board (IRB) approval and informed consent have been waived. Select the ADE reports related to “cancer” from the FAERS database since its establishment. Based on the FAERS database, with the keyword “cancer” (including those containing the character “cancer”, i.e., indications related to cancer, uniformly referred to as “cancer”) as the indication, extract the adverse events of drugs used to treat “cancer” from the establishment of the database to the present.

To comprehensively evaluate the safety of cancer drugs in clinical application, this study used “ipilimumab (including searches by different names and aliases such as Anti-CTLA-4 MAb, Anti CTLA 4 MAb ipilimumab, ipilimumab, Anti-CTLA-4 MAb, MDX 010, MDX-010, MDX010, MDX-CTLA-4, MDX CTLA 4, yervoy, and ipilimumab, which will be uniformly referred to as “ipilimumab” later), nivolumab (including searches by different names and aliases such as nivolumab and opdivo, which will be uniformly referred to as “nivolumab” later), and ipilimumab/nivolumab” as search keywords to extract data on ADEs from the first quarter (Q1) of 2004 to the fourth quarter (Q4) of 2024 from the FAERS database. The Preferred Terms (PT) and System Organ Classes (SOC) in the Medical Dictionary for Regulatory Activities (MedDRA) (v 21.0) (18) were adopted to describe and classify the relevant ADEs. The SOCs were grouped according to criteria such as etiology (such as infections and infestations), anatomical site (such as gastrointestinal disorders), and purpose (such as various surgical and medical procedures). A PT (Preferred Term) is a specific term used to express a single medical concept, such as a symptom, sign, disease, diagnosis, indication, examination, surgical or medical procedure, medical history, social history, or family history. Each PT corresponds to at least one SOC, and it can also correspond to multiple SOCs as appropriate.

In this study, according to the FDA-recommended method for removing duplicate reports, SAS software (v 9.4) (19) was used to sort the fields CASEID, FDA_DT, and PRIMARYID in the DEMO table of the downloaded ASCII data package. For reports with the same CASEID, the report with the most recent or latest FDA_DT value was retained; if both CASEID and FDA_DT fields were the same, the report with the largest PRIMARYID value was retained. After data deduplication, reports with CASEIDs listed in the deleted report list were excluded. ADE reports of the primary suspect drugs were then screened by matching DRUGNAME to the target drug names above. ADEs were classified and organized according to PT and SOC in the 21.0th edition of the MedDRA. Detailed prescribing information was obtained by querying the Food and Drug Administration Label (FDALabel) database (https://nctr-crs.fda.gov/fdalabel/ui/search). The drug roles of ADEs mainly included primary suspect (PS), secondary suspect drug (SS), concomitant (C), and interacting (I). During data processing, reports with the role code “PS” were included.

Data cleaning was carried out, and the data exclusion criteria were as follows: ① Duplicate items in DEMO, DRUG, REAC, THER, RPSR, and OUTC were deleted to improve the reliability of the results; ② Adverse event identifiers (IDs) for drug therapies of ipilimumab and nivolumab used in cancer that have been officially deleted, duplicated, or missing in the FDA were excluded; ③ Reports with missing information on gender, age, severity outcomes, and days to onset were excluded. All included data were processed.

### 2.2 Data mining

In this study, the disproportionality analysis was adopted to mine the ADE signals of “cancer” based on the terminology and classification defined by MedDRA version 21.0. Its basic principle was to use a disproportionality quadruple table to compare the differences in the occurrence frequencies of the target drug and the target event as well as the background frequencies. In the disproportionality analysis, four algorithms were used to detect ADEs signals: ROR, PRR, BCPNN, and EBGM. The specific formulas for the four algorithms were shown in **Supplementary Table 1**. The quadruple table of the disproportionality analysis was shown in **Supplementary Table 2**. Among them, CI was the confidence interval, χ2 was the chi-square, IC was the information component, IC025 was the lower limit of the 95% one-sided CI of IC, EBGM05 was the lower limit of the 95% one-sided CI of EBGM, a was the number of reports of ipilimumab, nivolumab, or ipilimumab/nivolumab accompanied by related adverse events, b was the number of reports of all other drugs accompanied by related adverse events, c was the number of reports of ipilimumab, nivolumab, or ipilimumab/nivolumab accompanied by all other adverse events, and d was the number of reports of all other drugs accompanied by all other adverse events. In this study, when the criteria of all four algorithms were simultaneously met, that is, for PRR: when PRR ≥ 2, χ2 ≥ 4 and a ≥ 3; for ROR: when the lower limit of the 95% CI of ROR > 1 and a ≥ 3; for BCPNN: when the lower limit of the 95% CI of IC (IC025) > 0; for EBGM: when EBGM > 2, the signal was determined as an effective ADE signal.

### 2.3 Statistical analysis

In this study, R software (v 4.2.3) (20) was used for statistical analysis and data processing. The dplyr package (v 1.1.4) (21) was applied to summarize the basic information of ipilimumab, nivolumab, and ipilimumab/nivolumab, and to characterize the ADEs observed in cancer treatment. To clarify the cumulative trends of ipilimumab, nivolumab, and ipilimumab/nivolumab ADEs and their onset timing across different age subgroups, the survival package (v 3.7.0) (22) was used to assess the median onset time of ADEs via survival curves. For ADE analysis at the SOC and PT levels, ROR, PRR, BCPNN, and EBGM methods were employed. Multivariate logistic regression analysis was performed for each drug using the survey package (v 4.4.1) (23) to explore the potential factors associated with reporting for ADEs and analyze the responses of specific patient subgroups (patients with fatal and non-fatal adverse reactions). The strength of association in ADE signal detection was determined using the ROR; the larger the ROR (95% CI), the higher the risk. Forest plots were constructed using the forestplot package (v 1.1.1) (24) for visual presentation (ROR (95% CI) > 1). Finally, the XGBoost package (v 2.0.3.1) (25) was used to evaluate the importance of the main factors in cancer treatment with ipilimumab, nivolumab, and ipilimumab/nivolumab.

To rigorously adjust for potential confounders and determine independent associations, a fully adjusted multivariate logistic regression model was subsequently constructed. Each positive adverse drug event signal (Preferred Term, PT) was treated as a binary outcome variable (occurrence vs. non-occurrence). The model simultaneously adjusted for the following covariates: (1) Demographic characteristics: age (continuous) and sex. Notably, body weight was excluded from this final model to prevent severe selection bias, as its missing data rate in the original dataset reached 60%–68%; (2) Concomitant medication burden: categorized as “0”, “1-2”, or “≥ 3” distinct concomitant drugs; (3) Cancer type (e.g., melanoma, non-small cell lung cancer [NSCLC], renal cell carcinoma [RCC], etc.); (4) Baseline disease burden: serving as a proxy for comorbidity burden, categorized by the number of non-cancer indications (“0”, “1”, or “≥ 2”); and (5) Organ dysfunction: a binary proxy variable (presence/absence) reflecting potential hepatic or renal impairment based on indication keywords. The models were fitted using binomial logistic regression to calculate adjusted odds ratios (aORs) and 95% confidence intervals (CIs). PTs with fewer than 10 events or models failing to converge were excluded to ensure analytical robustness.

## 3 Results

### 3.1 Baseline characteristics

This study included a total of 21,712,563 reports as study subjects, among which there were 4,600, 36,556, and 10,862 adverse reaction reports for ipilimumab, nivolumab, and ipilimumab/nivolumab, after excluding irrelevant data. A statistical analysis was conducted on the basic characteristics of the adverse events in cancer treatment (**Supplementary Tables S1**–**S3**). Among the three drug groups, the proportion of male (M) patients was relatively high (67.4%, 62.7%, and 63.4%, respectively), suggesting that male patients were more prone to adverse reactions. Patients weighing 50–100 kg accounted for a significant proportion (30.6%, 26.2%, and 32.1%, respectively), indicating that this weight range was associated with a higher risk of adverse reactions. Patients aged 65 to 85 years accounted for a significant proportion (49.3%, 40.8%, and 43.0%, respectively). Additionally, medical doctors (MD) comprised a high proportion of reporters (49.3%, 34.8%, and 44.1%, respectively). Japan accounted for a significant proportion of the reporting countries, at 58.7% (**Supplementary Table S1**); for nivolumab, the United States (US) accounted for a relatively high proportion of the reporting countries, at 35.0% (**Supplementary Table S2**); and for ipilimumab/nivolumab combination therapy, Japan also accounted for a relatively high proportion of the reporting countries, at 35.2% (**Supplementary Table S3**). In conclusion, based on FAERS baseline data for cancer treatment from Q1–2004 to Q4 2024, male patients who were aged 65 to 85 years and weighing 50–100 kg should be closely monitored for adverse reactions during treatment with ipilimumab, nivolumab, or their combination, and timely intervention is warranted.

### 3.2 Adverse reaction time analysis

**Figure 1:**
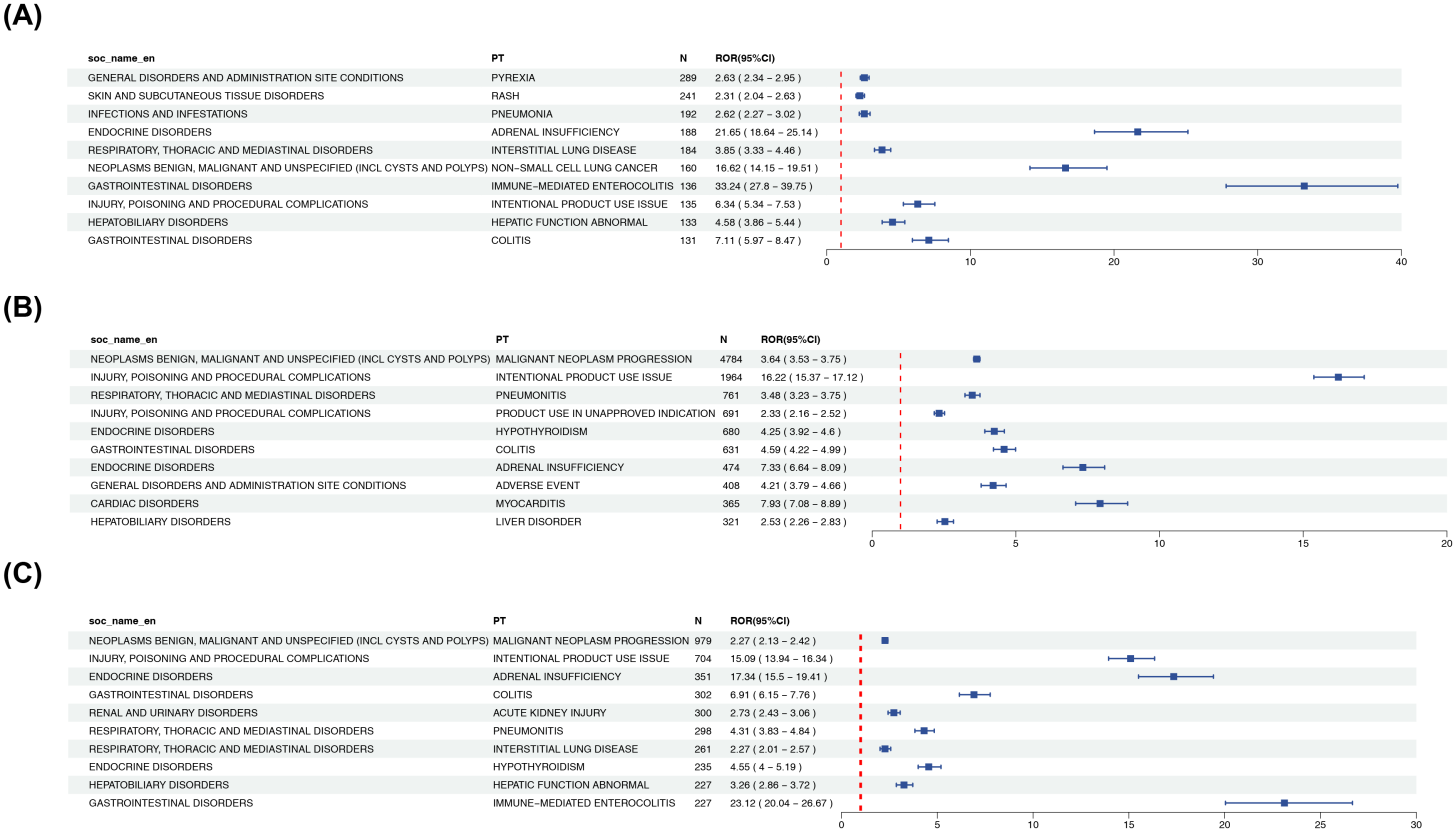
Median survival curves for adverse reaction onset time across different age groups. **(A)** Ipilimumab; **(B)** Nivolumab; **(C)** Ipilimumab/Nivolumab combination therapy.

The cumulative trends of adverse reaction occurrences across different age groups were presented, showing the median time and trends for the 3 drug groups. For ipilimumab, the trends across all age groups were relatively consistent except for patients under 18 years old and those aged 85 years and above. The cumulative percentage of adverse reactions rose rapidly within the first 30 days of treatment, indicating that adverse reactions were mainly concentrated in the early stage of treatment. Thereafter, the growth rate of the cumulative percentage slowed down gradually, suggesting that the incidence of adverse reactions decreased over time. The median time for all data of this drug was 43 days, meaning that approximately half of the patients experienced adverse reactions before day 43. This pattern was characterized by the bimodal features of “early concentrated outbreak + long-term sustained risk”. For patients under 18 years old, adverse reactions occurred before day 150 and no further adverse reactions were observed afterwards; similarly, in patients aged 85 years and above, adverse reactions appeared on day 270 with no subsequent occurrences (**Figure 1A**). For nivolumab, the trends across all age groups were relatively consistent, showing the bimodal pattern of “early concentrated outbreak + long-term sustained risk”. The median time for all data of this drug was 55 days. Among them, the median time for patients under 18 years old was 41.5 days, indicating that minor patients entered the long-term sustained risk phase earlier (**Figure 1B**). For ipilimumab/nivolumab combination therapy, the trends across all age groups were relatively similar, also presenting the bimodal pattern of “early concentrated outbreak + long-term sustained risk”. The median time for all data of this drug was 47 days. Among them, the median time for patients under 18 years old was 25.5 days, indicating that minor patients entered the long-term sustained risk phase earlier. Additionally, the median time for patients aged 85 years and above was 40 days, suggesting that elderly patients also entered the long-term sustained risk phase relatively early (**Figure 1C**). In conclusion, these results not only confirmed the consistency of the “early concentrated outbreak + long-term sustained risk” bimodal pattern across the 3 drug groups but also revealed age-related changes in reaction timing, emphasizing the necessity of personalized monitoring strategies to address the risks of acute and long-term adverse events in clinical practice.

### 3.3 Analysis of ADE based on PT levels

**Figure 2:**
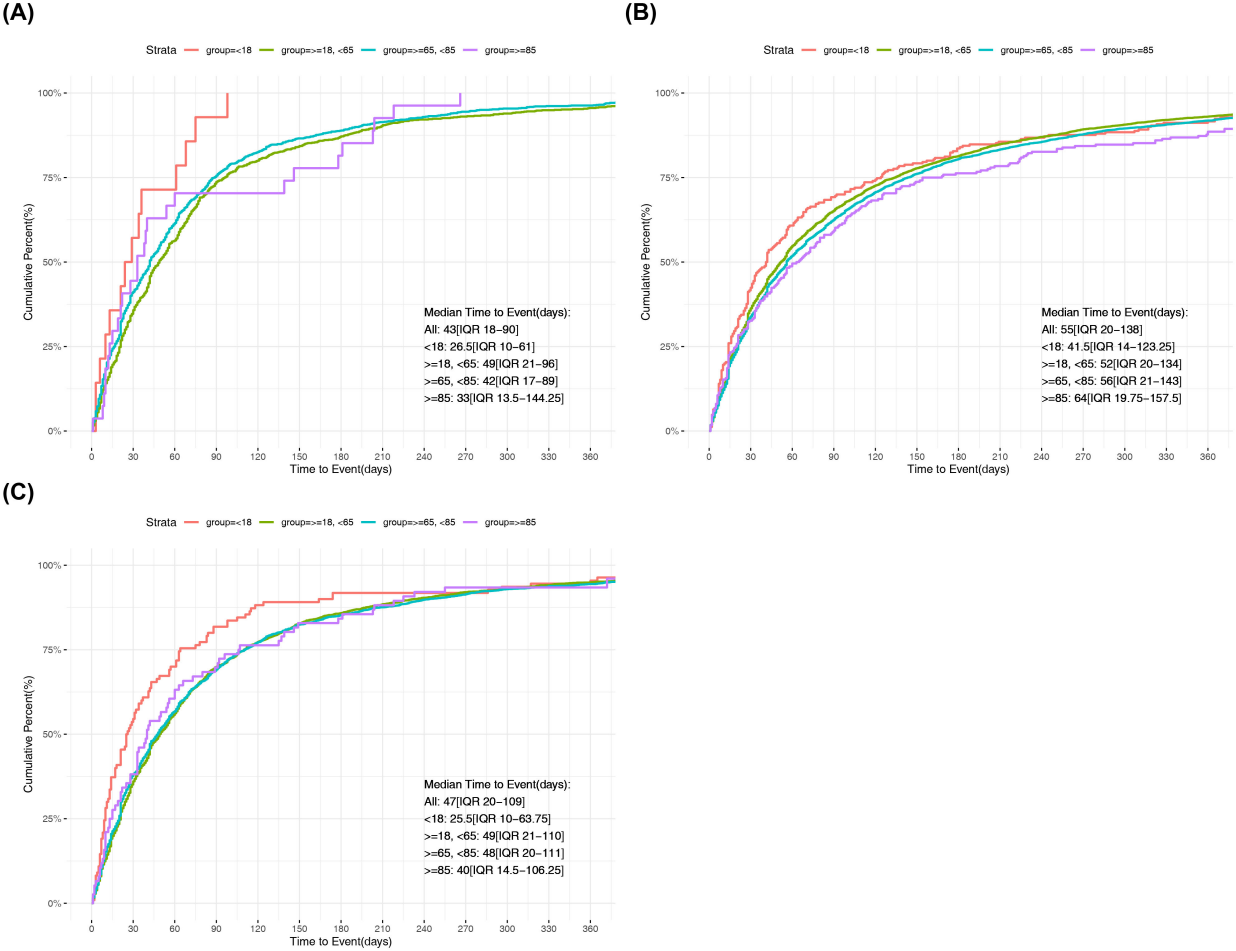
Risk forest plots from disproportionality analysis of adverse drug event (ADE) signals. **(A)** Ipilimumab; **(B)** Nivolumab; **(C)** Ipilimumab/Nivolumab combination therapy.

Based on the PT classification, a total of 1,443 cancer treatment-related ADEs were detected for ipilimumab, among which 191 were identified as positive signals. The top 10 ADEs sorted by the number of reports in descending order included adrenal insufficiency, non-small cell lung cancer, immune-mediated enterocolitis, etc. The ROR values of these adverse reactions were significantly higher than 1, indicating a strong association between these adverse reactions and ipilimumab. All these values met the criteria for identifying valid ADE signals in this study (number of ADE reports ≥ 3, lower limit of 95% CI of ROR > 1, PRR ≥ 2, χ² ≥ 4, IC025 > 0, EBGM > 2) (**Figure 2A**) (**Supplementary Table S4**). For nivolumab, a total of 4,308 cancer treatment-related ADEs were detected, among which 352 were identified as positive signals, such as malignant neoplasm progression, adrenal insufficiency, and myocarditis. These were significantly associated with nivolumab (**Figure 2B**) (**Supplementary Table S5**). For ipilimumab/nivolumab, a total of 2,386 cancer treatment-related ADEs were detected, with 253 confirmed as positive signals, including intentional product use issue, immune-mediated enterocolitis, and adrenal insufficiency. These showed a significant association with ipilimumab/nivolumab (**Figure 2C**) (**Supplementary Table S6**). Notably, all 3 drug groups involved colitis, adrenal insufficiency, malignant neoplasm progression, death, and intentional product use issue, indicating that these were significantly relevant ADE signals across the 3 groups. It should be noted that the term “intentional product use issue” primarily refers to medication errors and may represent coding misclassification within the FAERS database rather than true drug misuse. Furthermore, it must be explicitly noted that the prominent signals for malignant neoplasm progression and death detected in this analysis cannot distinguish between actual drug toxicity and the natural disease trajectory of advanced cancer. These specific outcomes are heavily confounded by disease severity, line of therapy, and subjective reporting practices. Therefore, they cannot be directly classified as adverse drug reactions; rather, they should be strictly viewed as descriptive reporting patterns within the database and interpreted with extreme caution.

### 3.4 Main factor analysis

**Figure 3:**
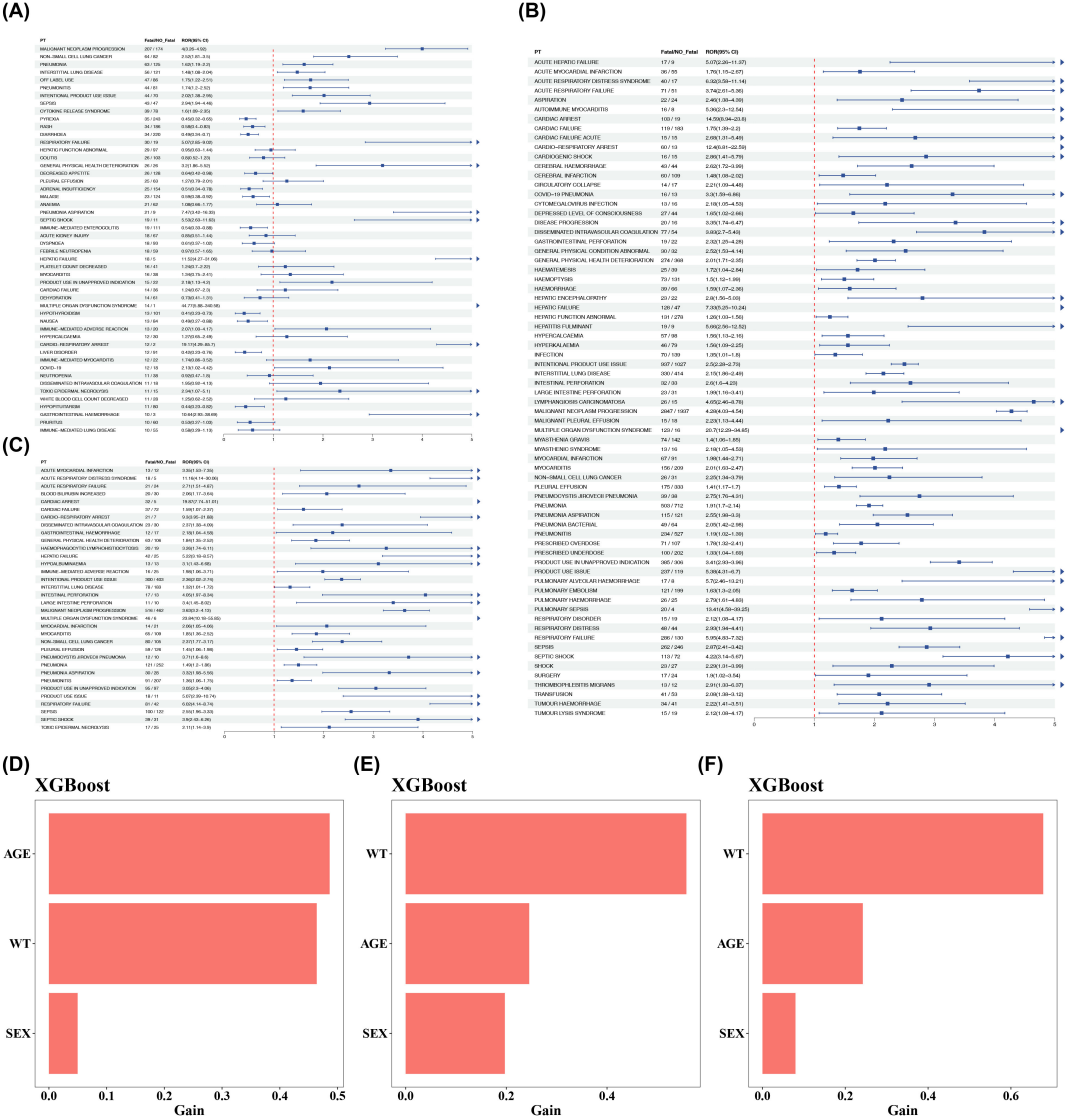
Risk factor analysis and assessment of main factor importance. **(A-C)** potential factors associated with reporting associated with death signals for Ipilimumab, Nivolumab, and Ipilimumab/Nivolumab, respectively. **(D-F)** Importance ranking of main factors for Ipilimumab, Nivolumab, and Ipilimumab/Nivolumab in cancer treatment, respectively.

In the analysis of potential factors associated with reporting, for ipilimumab, malignant neoplasm progression, general physical health deterioration, non-small cell lung cancer, sepsis, and hepatic failure were all significantly correlated with death signals (**Figure 3A**). For nivolumab, cardiac arrest, cardio-respiratory arrest, malignant neoplasm progression, acute respiratory distress syndrome, and hepatic failure were significantly associated with death signals (ROR > 1) (**Figure 3B**). For ipilimumab/nivolumab, cardiac arrest, hepatic failure, multiple organ dysfunction syndrome, respiratory failure, and septic shock were significantly correlated with death signals (ROR > 1) (**Figure 3C**). The XGBoost assessment showed that the importance of the main factors in cancer treatment with ipilimumab was in the order of age, weight, and gender (**Figure 3D**); for nivolumab in cancer treatment, the importance of the main factors was in the order of weight, age, and gender (**Figure 3E**); and for ipilimumab/nivolumab in cancer treatment, the importance of the main factors was in the order of weight, age, and gender (**Figure 3F**). In the present analysis, age and weight emerged as the top-ranking factors associated with adverse event reporting. However, given the limitations of the FAERS database (e.g., the lack of clinical covariates such as patient performance status, line of therapy, and underlying comorbidities), these findings must be interpreted strictly as hypothesis-generating associations within this reporting database, rather than validated independent clinical predictors. While enhanced clinical monitoring for elderly patients and those with extreme body weight might be considered, prospective studies are imperative for robust validation.

Following multivariate logistic regression adjustment, distinct yet highly consistent cross-drug reporting patterns emerged (**Supplementary Tables 1**-**6**). Across all three therapeutic groups, organ dysfunction was identified as the most potent and universally consistent factor positively associated with adverse event reporting, including mortality. Specifically, organ dysfunction significantly elevated the reporting odds of death for ipilimumab (aOR = 3.20, P = 0.005), nivolumab (aOR = 2.37, P = 6.69 × 10^-11^), and the combination therapy (aOR = 1.80, P = 0.027). For ipilimumab monotherapy, 43 PTs demonstrated significant adjusted associations, with organ dysfunction exhibiting a median aOR of 7.3. For nivolumab monotherapy, 148 PTs showed significant associations, with organ dysfunction again acting as the most stable associated factor (median aOR = 5.55). Conversely, a higher concomitant medication burden (e.g., ≥ 3 drugs) and specific solid tumor types (such as NSCLC and RCC) generally exhibited an inverse (protective) association with adverse event reporting and mortality across the cohorts. Baseline disease burden consistently showed an inverse association with mortality endpoints but demonstrated positive associations with specific non-fatal adverse events. These findings confirm that after controlling for demographics, concomitant medications, cancer types, and comorbidities, organ dysfunction remains a critical, cross-drug factor associated with elevated reporting of severe adverse events.

## 4 Discussion

The therapeutic landscape of oncology has witnessed substantial expansion in immune checkpoint inhibitor utilization. Ipilimumab and nivolumab, which respectively target CTLA-4 and PD-1 signaling pathways, potentiate immune activation against malignant cells (26, 27). Accumulating clinical evidence substantiates the efficacy of this combinatorial approach across diverse malignancies (28, 29), including recurrent or metastatic head and neck squamous cell carcinoma, where it demonstrates significant overall survival benefit (30). Notwithstanding these therapeutic advantages, combination regimens exhibit heightened incidence and diversity of immune-related adverse events relative to monotherapeutic approaches (9).

The recognition of immunotherapy-associated cardiotoxicity (IAC) as a distinct toxicity class has driven a marked increase in reported prevalence over the past biennium (31). Previous pharmacovigilance investigations utilizing the FAERS database have identified crizotinib-associated unexpected significant adverse events, including deep vein thrombosis, Pneumocystis jirovecii pneumonia, gastrointestinal amyloidosis, and hepatic coma (32). Similarly, a separate FAERS analysis of ipilimumab-nivolumab safety profiles detected 125 unanticipated significant adverse events (9). However, a comparative safety assessment of monotherapeutic versus combination regimens remains unaddressed in the current literature.

This investigation comprehensively characterized adverse drug event (ADE) profiles for ipilimumab monotherapy, nivolumab monotherapy, and their combination regimen in oncological practice, leveraging FAERS data from Q1–2004 through Q4 2024. Methodologically, we delineated baseline characteristics of adverse reaction reports, conducted temporal trend analyses for each therapeutic category, performed PT-level ADE mining, and identified risk determinants for adverse reactions. These findings offer valuable clinical insights for high-risk population identification and monitoring schedule optimization.

Through systematic analysis of baseline characteristics and potential factors associated with reporting, this investigation established age and weight as significant risk determinants across all three therapeutic modalities. Specifically, patients aged 65–85 years, weighing 50–100 kg, and male gender require heightened clinical vigilance. These demographic parameters warrant further investigation regarding their relationship with treatment outcomes. Consistent with our findings, existing literature suggests that elderly patients, specific cancer subtypes, and dual ICI regimens correlate with elevated risk of fatal neurological adverse events (33). Large-scale FAERS analyses demonstrate that patients ≥65 years exhibit significantly heightened overall risk of immune-related adverse events (irAEs) following ICI administration compared to younger cohorts (<65 years). Among ICIs, ipilimumab (CTLA-4 inhibitor) demonstrates the highest irAE risk in elderly populations relative to PD-1/PD-L1 inhibitors, with ipilimumab-nivolumab combination therapy carrying the greatest risk (34). These observations align with our conclusion regarding age as a significant risk factor. Population pharmacokinetic characterization of ipilimumab reveals that drug clearance correlates positively with body weight and baseline serum lactate dehydrogenase concentrations (35). Consequently, individualized risk assessment and enhanced monitoring for elderly patients and specific weight cohorts are imperative for optimizing immunotherapeutic safety management.

Although XGBoost and multivariate logistic regression identified age and weight as the variables most strongly associated with adverse event reporting, these results demand cautious interpretation. First, within a spontaneous reporting system like FAERS, demographic variables such as age and weight frequently serve as proxies for unmeasured confounders, including performance status, tumor burden, comorbidity indices, treatment eligibility, or cachexia (36). Second, the feature importance rankings derived from machine learning algorithms exhibit inherent dataset specificity and may not broadly generalize to diverse populations or alternative clinical environments (37). Third, the absence of crucial covariates (e.g., history of autoimmune diseases, baseline corticosteroid use, and specific cancer types) introduces inevitable residual confounding (38). Consequently, these findings must be viewed exclusively as descriptive associations within the reported cases, rather than validated, patient-level risk stratification tools. Future prospective investigations incorporating comprehensive clinical datasets are required to ascertain the true predictive value of age and weight for immune checkpoint inhibitor-related adverse events.

Our FAERS analysis revealed a distinct bimodal temporal distribution of adverse event onset—characterized by early concentration and prolonged persistence—with significant inter-age-group variation in median time to onset. The cumulative incidence of ipilimumab-associated adverse events increased rapidly within the initial 30-day treatment period, identifying this interval as a high-risk window for acute toxicities. This pattern corresponds to the pharmacological mechanism of ICIs acting during early immune activation phases, wherein rapid T-cell activation may precipitate acute immune-mediated injury in cutaneous and gastrointestinal tissues (39).

When comparing the three therapeutic regimens directly, the median onset time for ipilimumab monotherapy was 43 days, compared to 55 days for nivolumab monotherapy and 47 days for the combination therapy. Although the absolute differences in median onset times across the three groups are relatively narrow (ranging from 43 to 55 days), ipilimumab and the combination regimen exhibited shorter median onset times relative to nivolumab alone. Clinically, the earlier onset associated with ipilimumab (43 days) and the combination therapy (47 days) suggests that CTLA-4 blockade, whether administered alone or in combination, is correlated with a more rapid manifestation of adverse events. This observation aligns with the established pharmacology of ipilimumab, which shows a signal for a broader and typically more acute systemic immune activation (40). From a clinical monitoring perspective, these findings imply that patients receiving ipilimumab-containing regimens may require more intensive surveillance during the initial 4 to 6 weeks of treatment, whereas the slightly delayed peak for nivolumab monotherapy might permit a modified monitoring schedule. Nevertheless, considering potential overlapping confidence intervals and substantial inter-individual variability, these temporal differences should be interpreted as a complementary tool for individualized risk assessment rather than a definitive substitute for standard clinical vigilance. Following the initial peak, the cumulative incidence curve progression decelerated but persisted, indicating sustained immune activation and potential long-term immune-related injuries necessitating extended surveillance. Notably, the combination therapy cohort exhibited the shortest median onset time (25.5 days) in patients <18 years, potentially reflecting heightened sensitivity to ICI-induced immune activation in immunologically immature populations. While ipilimumab monotherapy in patients >85 years demonstrated no new adverse events beyond 270 days, the median onset time in the combination therapy subgroup (40 days) preceded that of the overall population (47 days), suggesting accelerated long-term risk manifestation in elderly patients receiving combination therapy, potentially attributable to age-related immune dysfunction and diminished organ reserve (41, 42).

PT-level analysis identified colitis, adrenal insufficiency, malignant neoplasm progression, mortality, myocarditis, and immune-mediated enterocolitis as significantly associated with the investigated therapeutic regimens. Existing literature documents similar irAE profiles in nivolumab-ipilimumab treated metastatic renal cell carcinoma patients, including myocarditis, colitis, and hepatitis (17). Melanoma therapies demonstrating highest adverse event incidence include ipilimumab, nivolumab, dabrafenib, and vemurafenib, with pyrexia (12.9%), rash (9.8%), diarrhea (9.5%), fatigue (7.0%), and colitis (6.7%) representing the most frequently reported events (43). Additional investigations confirm that nivolumab-ipilimumab combination therapy significantly elevates risks for gastrointestinal, endocrine, hepatobiliary, metabolic, nutritional, and respiratory disorders, particularly colitis, hypophysitis, pneumonia, and hepatitis (10).

A critical aspect of this pharmacovigilance analysis is the identification of endocrine-specific adverse events, which is particularly relevant to the evolving field of cancer endocrinology. It is well-established that immune checkpoint inhibitors can trigger a wide spectrum of endocrine immune-related adverse events (irAEs), including hypophysitis, thyroiditis, adrenal insufficiency, and type 1 diabetes mellitus (44). The present analysis demonstrates a significantly high reporting trend for adrenal insufficiency across all three treatment groups, aligning with previous clinical observations. Signals for hypophysitis and thyroid dysfunction were also detected, appearing more frequently in ipilimumab-containing regimens. These endocrine irAEs bear profound clinical significance, as they frequently necessitate lifelong hormone replacement therapy and are largely irreversible. The underlying pathogenesis involves autoimmune-mediated attacks on endocrine glands, with CTLA-4 inhibitors (such as ipilimumab) precipitating such events at a notably higher frequency than PD-1 inhibitor monotherapy (45). Consequently, these findings strongly emphasize the absolute necessity of rigorous endocrinological monitoring for patients undergoing these immunotherapies. Such surveillance must encompass comprehensive baseline evaluations and periodic assessments of thyroid function, morning cortisol levels, and blood glucose concentrations. Furthermore, clinicians must remain highly vigilant that adrenal insufficiency may initially present with non-specific symptoms—such as fatigue, nausea, and hypotension—which, if unrecognized and unmanaged, can rapidly progress to life-threatening adrenal crises (46). Notably, ipilimumab-related colitis represents the most frequently reported ADE, while nivolumab-associated myocarditis predominates among cardiotoxicities (47). Substantial variability exists in immune-mediated enterocolitis risk across ICI classes, with CTLA-4 inhibitors (particularly tremelimumab and ipilimumab) demonstrating strongest association (48). Collectively, these findings underscore the necessity for enhanced adverse reaction signal detection and preemptive preventive measures in oncological immunotherapy.

Notably, a strong signal for “intentional product use issue” was detected across all three treatment groups. In the MedDRA dictionary, this term is classified under medication errors and administration issues, which are distinct from typical immune-related adverse events (irAEs). Given the clinical context of immune checkpoint inhibitor administration, the likelihood of genuine intentional misuse or overdose is extremely low. The high frequency of this signal is more likely attributable to coding biases inherent in the spontaneous reporting system of FAERS. Reporters from diverse backgrounds, including non-healthcare professionals, might inaccurately assign any product-related administration issues to this Preferred Term (PT) due to unfamiliarity with MedDRA terminology. Furthermore, severe irAEs or critical clinical events might have been erroneously misclassified under this term during the reporting process. Therefore, this specific result requires cautious interpretation and does not indicate widespread clinical drug misuse. This finding highlights the necessity of prioritizing the evaluation of coding biases when analyzing medication error-related terms in the FAERS database, rather than accepting them immediately as true adverse events (49). It also underscores the importance of standardized MedDRA terminology application among reporters to minimize the misclassification of severe irAEs.

In the present study, positive disproportionality signals for malignant neoplasm progression and death were detected across all three treatment groups. However, these findings must be interpreted with extreme caution. Within the FAERS database, the population receiving ipilimumab or nivolumab predominantly consists of patients with advanced or metastatic cancers, a demographic inherently possessing an exceptionally high baseline risk of disease progression and mortality. Due to the inherent lack of granular data regarding tumor stage, line of therapy, patient performance status, and specific causes of death, it is impossible to differentiate whether these reports reflect drug-attributable toxicities (such as fatal immune-related adverse events) or merely the natural progression of the underlying malignancies (50). Furthermore, substantial reporting biases likely exist; clinicians may be disproportionately inclined to report any severe clinical outcome, including progression or death, for patients undergoing novel immunotherapies (51). Consequently, these signals absolutely should not be construed as evidence that ipilimumab or nivolumab accelerates cancer progression or increases overall mortality. Instead, they must be reframed as descriptive “reporting patterns” that underscore the necessity of rigorous pharmacovigilance in this critically ill population. Future research employing active comparator designs or target trial emulation methods is required to accurately evaluate the true drug-attributable risks for these hard clinical endpoints (52).

This investigation collated adverse reaction data for ipilimumab, nivolumab, and their combination regimen from FAERS (2004–2024), analyzing temporal cumulative incidence trends and employing disproportionality analysis to identify safety signals in oncological applications. Furthermore, we characterized potential factors associated with reporting for treatment-emergent adverse events, providing substantiative support for clinical monitoring and molecular potential mechanism research. Several limitations warrant acknowledgment: the inherent reporting bias and underreporting characteristics of spontaneous reporting systems like FAERS; the unpredictable nature of real-world adverse reactions despite rigorous pre-market clinical testing; and the statistical rather than clinical significance of detected signals, necessitating subsequent clinical validation. Future investigations characterizing ADE profiles when immunotherapies are combined with conventional modalities (chemotherapy, targeted therapy) would yield additional clinical insights. The multivariate logistic regression analysis provided critical insights into the complex interplay of clinical covariates. The emergence of organ dysfunction as the most robust factor associated with elevated reporting of ADEs and mortality across all three regimens highlights the vulnerability of patients with pre-existing hepatic or renal impairment to severe immunotherapies-related toxicities. Interestingly, a higher concomitant medication burden and specific cancer types (e.g., NSCLC, RCC) demonstrated inverse (protective) associations with ADE reporting. Rather than implying true biological protection, this phenomenon likely reflects a “reporting dilution effect” inherent to spontaneous pharmacovigilance databases; patients with complex medication regimens or advanced specific cancers may experience competing risks or overlapping toxicities, making clinicians less likely to attribute a specific adverse event solely to the immune checkpoint inhibitor. Additionally, the exclusion of body weight from the multivariate model due to severe missingness underscores the pervasive issue of data incompleteness in FAERS, reaffirming that these statistical associations should inform clinical vigilance—particularly for patients with organ dysfunction—rather than serve as absolute predictive tools. Furthermore, the FAERS database possesses inherent methodological limitations regarding causal inference; spontaneous reporting systems can only detect associative signals rather than establish definitive causal relationships. Additionally, the database lacks granular clinical details concerning disease severity (e.g., tumor stage, performance status) and concomitant therapies (e.g., chemotherapy, radiotherapy, targeted agents, or supportive care). Given that disease severity and concurrent treatments act as major drivers for both drug administration and adverse event reporting, their absence introduces potential confounding biases that cannot be fully adjusted for in the current analysis. Therefore, the present findings should be strictly interpreted as hypothesis-generating and require further validation through well-controlled prospective clinical studies.

## Data Availability

The original contributions presented in the study are included in the article/**Supplementary Material**. Further inquiries can be directed to the corresponding authors.

## Supplementary material

Supplementary Table 1 Full multivariable logistic regression analysis of potential factors associated with all analyzed adverse events (PT level) in patients receiving ipilimumab monotherapy.

Supplementary Table 2 Statistically significant associations derived from multivariable logistic regression analysis for adverse events (PT level) in patients receiving ipilimumab monotherapy.

Supplementary Table 3 Full multivariable logistic regression analysis of potential factors associated with all analyzed adverse events (PT level) in patients receiving ipilimumab and nivolumab combination therapy.

Supplementary Table 4 Statistically significant associations derived from multivariable logistic regression analysis for adverse events (PT level) in patients receiving ipilimumab and nivolumab combination therapy.

Supplementary Table 5 Full multivariable logistic regression analysis of potential factors associated with all analyzed adverse events (PT level) in patients receiving nivolumab monotherapy.

Supplementary Table 6 Statistically significant associations derived from multivariable logistic regression analysis for adverse events (PT level) in patients receiving nivolumab monotherapy.
